# Laparo-endoscopic versus open recurrent inguinal hernia repair: should we follow the guidelines?

**DOI:** 10.1007/s00464-016-5342-7

**Published:** 2016-12-08

**Authors:** F. Köckerling, R. Bittner, A. Kuthe, B. Stechemesser, R. Lorenz, A. Koch, W. Reinpold, H. Niebuhr, M. Hukauf, C. Schug-Pass

**Affiliations:** 1 Department of Surgery and Center for Minimally Invasive Surgery, Academic Teaching Hospital of Charité Medical School, Vivantes Hospital, Neue Bergstrasse 6, 13585 Berlin, Germany; 2 Hernia Center, Winghofer Medicum, Winghofer Strasse 42, 72108 Rottenburg am Neckar, Germany; 3 Department of General and Visceral Surgery, German Red Cross Hospital, Lützerodestrasse 1, 30161 Hannover, Germany; 4 Hernia Center Cologne, PAN – Hospital, Zeppelinstrasse 1, 50667 Cologne, Germany; 5 3Surgeons, Klosterstrasse 34/35, 13581 Berlin, Germany; 6 Hernia Center Cottbus, Gerhard-Hauptmann-Strasse 15, 03044 Cottbus, Germany; 7 Department of Surgery and Hernia Center, Wilhelmsburg Hospital Gross-Sand, Gross-Sand 3, 21107 Hamburg, Germany; 8 Hanse-Hernia Center, Alte Holstenstrasse 16, 21031 Hamburg, Germany; 9 StatConsult GmbH, Halberstädter Strasse 40 a, 39112 Magdeburg, Germany

**Keywords:** Inguinal hernia, Recurrence, Postoperative complications, Pain, Endoscopic repair

## Abstract

**Introduction:**

On the basis of six meta-analyses, the guidelines of the European Hernia Society (EHS) recommend laparo-endoscopic recurrent repair following previous open inguinal hernia operation and, likewise, open repair following previous laparo-endoscopic operation. So far no data are available on implementation of the guidelines or for comparison of outcomes. Besides, there are no studies for comparison of outcomes for compliance versus non-compliance with the guidelines.

**Patients and methods:**

In total, 4812 patients with elective unilateral recurrent inguinal hernia repair in men were enrolled between September 1, 2009, and September 17, 2014, in the Herniamed Registry. Only patients with 1-year follow-up were included.

**Results:**

Out of the 2482 laparo-endoscopic recurrent repair operations 90.5% of patients, and out of the 2330 open recurrent repair procedures only 38.5% of patients, were operated on in accordance with the guidelines of the EHS. Besides, on compliance with the guidelines multivariable analysis demonstrated for laparo-endoscopic recurrent repair a significantly lower risk of pain at rest (OR 0.643 [0.476; 0.868]; *p* = 0.004) and pain on exertion (OR 0.679 [0.537; 0.857]; *p* = 0.001). Comparison of laparo-endoscopic and open recurrent repair in settings of compliance versus non-compliance with the guidelines showed a higher incidence of perioperative complications and re-recurrences for recurrent repairs that did not comply with the guidelines.

**Conclusion:**

The EHS guidelines for recurrent inguinal hernia repair are not yet being observed to the extent required. Non-compliance with the guidelines is associated with higher perioperative complication rates and higher risk of re-recurrence. Even on compliance with the guidelines, the risk of pain at rest and pain on exertion is higher after open recurrent repair than after laparo-endoscopic repair.

Compared with primary inguinal hernia operations, both open and laparo-endoscopic recurrent repair procedures are associated with a higher rate of perioperative complications, re-recurrences and chronic pain [1, 2]. Six meta-analyses are available for comparison of laparo-endoscopic with open recurrent inguinal hernia repairs [3–8]. These meta-analyses analyzed 12 studies [9–20]. Compared with the meta-analysis by Li et al. [7], which included non-randomized studies [12, 13, 16, 19], the meta-analysis by Pisanu et al. [6] featured the largest number of exclusively prospective randomized studies [9, 11, 14, 15, 17, 18, 20]. There was no high risk of bias in any of the included trials [6]. The studies included in total 647 patients with recurrent inguinal hernia randomized to either laparo-endoscopic repair [*n* = 333; 51.5%, transabdominal preperitoneal patch plasty (TAPP) and totally extraperitoneal patch plasty (TEP)], or anterior open repair (*n* = 314; 48.5%, by Lichtenstein technique). Patients who underwent laparo-endoscopic repair experienced significantly less chronic pain (9.2 vs 21.5%; *p* = 0.003). Patients of the laparo-endoscopic group had a significantly earlier return to normal daily activities (13.9 vs 18.4 days, SMD −0.68, 95% CI −0.94 to −0.43; *p* < 0.000001). Operative time was significantly longer in laparo-endoscopic operations (62.9 vs 54.2 min, SMD 0.46, 95% CI 0.03, 0.89; *p* = 0.04) [6]. No other differences were found [6]. Another prospective randomized controlled study that was not included in the meta-analyses also identified a lower chronic pain rate after laparo-endoscopic recurrent repair [21]. A Swedish registry study likewise demonstrated on comparing anterior mesh repair with laparo-endoscopic mesh repair for recurrent hernias a lower risk of chronic pain for the laparo-endoscopic operation (OR 0.54 [CI 0.30–0.97]; *p* = 0.039) [22].

On the basis of the meta-analyses, the European Hernia Society recommends laparo-endoscopic inguinal hernia repair of recurrent hernias after conventional open repair [8, 23] and for recurrent hernias after laparo-endoscopic hernia repair an open procedure. Likewise, the International Endohernia Society recommends, with a high level of evidence, TEP and TAPP for repair of recurrent hernia as the preferred alternative to tissue repair and to the Lichtenstein repair after prior anterior repair [24, 25]. In the Consensus Development Conference of the European Association of Endoscopic Surgery, TEP and TAPP are preferred in patients with a recurrent groin hernia after open repair. Repeat endoscopic repair is only feasible when the surgeon has a high level of experience in repeat endoscopic groin hernia repair [26]. However, registry data show that even following previous open suture and mesh repair to treat the primary inguinal hernia, open suture and mesh repair are used once again for a recurrent hernia [27]. That is due to the fact that the skill needed for laparo-endoscopic recurrent inguinal hernia repairs was not always assured. Where surgeons had used an open technique to repair 95% of primary inguinal hernias, then more than 90% of recurrences were also repaired using an open procedure [28]. That was also true when using mesh repair for the primary inguinal hernia operation [13].

This present analysis of data from the Herniamed Hernia Registry [29] now investigates: (1) To what extent surgeons implement the guidelines of the international hernia societies. (2) Since to date no study has compared the outcomes of open and laparo-endoscopic recurrent inguinal hernia repair carried out in compliance with the guidelines, that aspect will now also be explored in the present analysis. (3) Finally, how the outcomes of open and laparo-endoscopic recurrent inguinal hernia repair differ on compliance versus non-compliance with the guidelines.

## Patients and methods

The Herniamed Registry is a multicenter, Internet-based hernia registry [29] into which 427 participating hospitals and surgeons engaged in private practice (Herniamed Study Group) have entered data prospectively on their patients who had undergone routine hernia surgery and signed an informed consent to participate. All postoperative complications occurring up to 30 days after surgery are recorded. On 1-year follow-up, postoperative complications are once again reviewed when the general practitioner and patient complete a questionnaire. Information is also obtained on any recurrence, pain at rest and on exertion as well as pain requiring treatment. This present analysis compares the prospective data collected for all male patients with a minimum age of 16 years who had undergone elective recurrent unilateral inguinal hernia repair using either transabdominal preperitoneal patch plasty (TAPP), total extraperitoneal patch plasty (TEP) or open repair in Lichtenstein, Should ice, TIPP and Plug techniques.

**Fig. 1:**
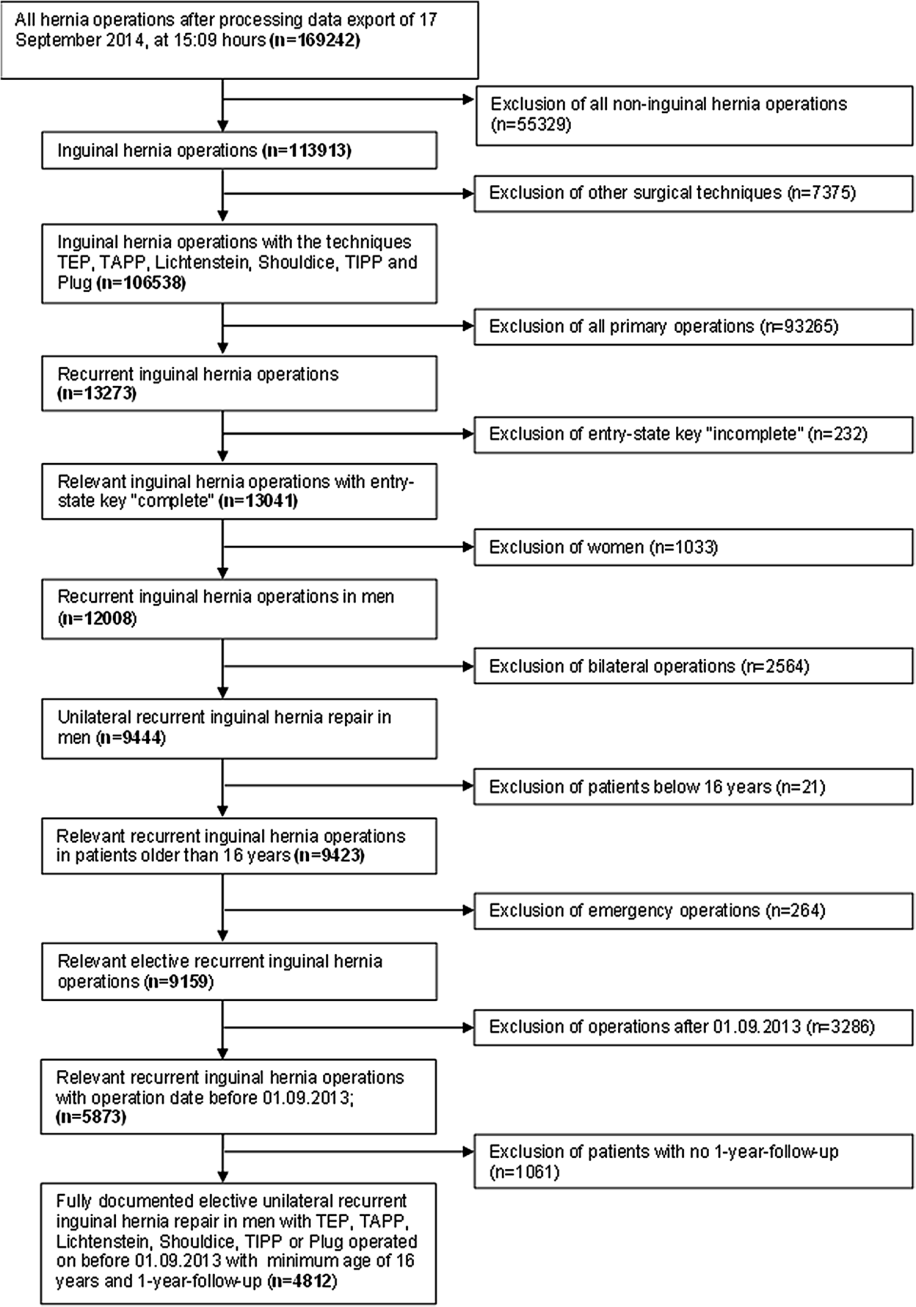
Flowchart of patient inclusion

In total, 4812 patients were enrolled between September 1, 2009, and August 31, 2013 (Fig. 1). Of these patients, 2482 (51.58%) had laparo-endoscopic and 2330 (48.42%) open repair. All the patients had to have a 1-year follow-up (follow-up rate 100%).

The demographic and surgery-related parameters included age (years), BMI (kg/m^2^), ASA classification (I, II, III–IV) as well as EHS classification (hernia type: medial, lateral, femoral, scrotal and defect size: grade I = <1.5 cm, grade II = 1.5–3 cm, grade III = >3 cm) [30] and general risk factors (nicotine, COPD, diabetes, cortisone, immunosuppression, etc.). Risk factors were dichotomized, i.e., ‘yes’ if at least one risk factor is positive and ‘no’ otherwise.

The dependent variables were intra- and postoperative complication rates, number of reoperations due to complications as well as the 1-year results (recurrence rate, pain at rest, pain on exertion and pain requiring treatment).

All analyses were performed with the software 9.2 (SAS 9.2 Institute Inc. Cary, NY, USA) and intentionally calculated to a full significance level of 5%, i.e., they were not corrected in respect of multiple tests, and each *p* value ≤0.05 represents a significant result. To discern differences between the groups in unadjusted analyses, Fisher’s exact test was used for categorical outcome variables and the robust t-test (Satterthwaite) for continuous variables.

To rule out any confounding of data caused by different patient characteristics, the results of unadjusted analyses were verified via multivariable analyses in which, in addition to laparo-endoscopic or open operation, other influence parameters were simultaneously reviewed.

To identify influence factors in multivariable analyses, the binary logistic regression model for dichotomous outcome variables was used. Estimates for odds ratio (OR) and the corresponding 95% confidence interval based on the Wald test were given. For influence variables with more than two categories, one of the latter forms was used in each case as reference category. For age (years) the 10-year OR estimate and for BMI (kg/m^2^) the five-point OR estimate were given. Results were presented in tabular form, sorted by descending impact.

## Results

1. To what extent do surgeons follow the guidelines?

In the laparo-endoscopic recurrent operation group, the recurrent operation was performed for *n* = 1528/2482 (61.6%) patients following the open suture technique for *n* = 718/2482 (28.9%) after open mesh repair, and for *n* = 233/2482 (9.4%) following laparo-endoscopic primary mesh repair (unknown 0.1%).

Open recurrent repair was performed for *n* = 1011/2330 (43.4%) patients following previous open suture repair, for *n* = 897/2330 (38.5%) patients following laparo-endoscopic mesh repair and for 412/2330 (17.7%) patients after open mesh repair of the primary inguinal hernia (unknown 0.4%).

Accordingly, in the laparo-endoscopic recurrent repair group 90.5%, and in the open recurrent repair group 38.5%, of patients were operated on in compliance with the guidelines of the international hernia societies.

2. Is there a difference in the outcome of open versus laparo-endoscopic recurrent inguinal hernia repair in compliance with the guidelines?

This analysis is based on *n* = 2246 laparo-endoscopic recurrent inguinal hernia repair operations following previous open primary operation and *n* = 897 open recurrent inguinal hernia repair operations following previous laparo-endoscopic primary repair (Table 1). Unadjusted analysis did not find any significant difference in the mean age between the two groups; however, the mean BMI value was higher for those patients undergoing open recurrent repair (Table 2). The open recurrent repair was associated with significantly larger hernia defects, more medial, fewer femoral and lateral EHS classifications (Table 3). No differences were identified in the risk factors (Table 3). Non-adjusted analysis of the target variables revealed that the intraoperative complications entailed more nerve injuries for open recurrent repair as well as more pain at rest and pain on exertion on 1-year follow-up (Table 4). No significant difference was detected between the laparo-endoscopic and open technique on performing recurrent repair in compliance with the guidelines for the following: overall intraoperative complication rate, postoperative complication rate, complication-related reoperation rate, recurrence rate and the rate of chronic pain requiring treatment.

**Table 1:** Recurrent operations according to the guidelines and previous operations

	Previous operations								Total	
	Unknown		Suture		Open mesh		Endoscopic mesh			
	*N*	%	*N*	%	*N*	%	*N*	%	*N*	%
Recurrent operation										
Endoscopic	3	0.1	**1528**	**61.6**	**718**	**28.9**	233	9.4	2482	100.0
Open	10	0.4	1011	43.4	412	17.7	**897**	**38.5**	2330	100.0
Total	13	0.3	2539	52.8	1130	23.5	1130	23.5	4812	100.0

Bold numbers are the operations in accordance with the guidelines

**Table 2:** Age and BMI of patients with laparo-endoscopic versus open unilateral recurrent inguinal hernia repair in men according to the guidelines

		Operation		*p*
		Endoscopic	Open	
Age (years)	Mean ± STD	58.9 ± 15.6	59.3 ± 15.3	0.440
BMI (kg/m^2^)	Mean ± STD	25.9 ± 3.4	26.3 ± 3.6	0.004

**Table 3:** Demographic and surgery-related parameters and risk factors for patients with laparo-endoscopic versus open unilateral recurrent inguinal hernia repair in men according to the guidelines

		Endoscopic		Open		*p*
		*n*	%	*n*	%	
ASA score	I	561	24.98	257	28.65	0.091
	II	1302	57.97	502	55.96	
	III/IV	383	17.05	138	15.38	
Defect size	I (<1.5 cm)	417	18.57	151	16.83	<0.001
	II (1.5–3 cm)	1459	64.96	493	54.96	
	III (>3 cm)	370	16.47	253	28.21	
EHS-classification medial	Yes	1112	49.51	518	57.75	<0.001
	No	1134	50.49	379	42.25	
EHS-classification lateral	Yes	1351	60.15	452	50.39	<0.001
	No	895	39.85	445	49.61	
EHS-classification femoral	Yes	77	3.43	15	1.67	0.007
	No	2169	96.57	882	98.33	
EHS-classification scrotal	Yes	27	1.20	12	1.34	0.724
	No	2219	98.80	885	98.66	
Risk factor						
Total	Yes	687	30.59	275	30.66	0.966
	No	1559	69.41	622	69.34	
COPD	Yes	151	6.72	66	7.36	0.534
	No	2095	93.28	831	92.64	
Diabetes	Yes	129	5.74	51	5.69	1.000
	No	2117	94.26	846	94.31	
Aortic aneurism	Yes	16	0.71	4	0.45	0.467
	No	2230	99.29	893	99.55	
Immunosuppression	Yes	14	0.62	10	1.11	0.174
	No	2232	99.38	887	98.89	
Corticoids	Yes	20	0.89	8	0.89	1.000
	No	2226	99.11	889	99.11	
Smoking	Yes	262	11.67	110	12.26	0.669
	No	1984	88.33	787	87.74	
Coagulopathy	Yes	33	1.47	9	1.00	0.390
	No	2213	98.53	888	99.00	
Antiplatelet medication	Yes	202	8.99	79	8.81	0.890
	No	2044	91.01	818	91.19	
Anticoagulation therapy	Yes	44	1.96	25	2.79	0.177
	No	2202	98.04	872	97.21

**Table 4:** Intra- and postoperative complications, complication-related reoperations and 1-year follow-up results of patients with laparo-endoscopic versus open unilateral recurrent inguinal hernia repair in men according to the guidelines

		Endoscopic		Open		*p*
		*n*	%	*n*	%	
Intraoperative complication						
Total	Yes	26	1.16	14	1.56	0.380
	No	2220	98.84	883	98.44	
Bleeding	Yes	15	0.67	3	0.33	0.431
	No	2231	99.33	894	99.67	
Injuries						
Total	Yes	17	0.76	12	1.34	0.147
	No	2229	99.24	885	98.66	
Vascular	Yes	8	0.36	0	0.00	0.115
	No	2238	99.64	897	100.0	
Bowel	Yes	5	0.22	0	0.00	0.330
	No	2241	99.78	897	100.0	
Bladder	Yes	2	0.09	1	0.11	1.000
	No	2244	99.91	896	99.89	
Nerve	Yes	0	0.00	9	1.00	<0.001
	No	2246	100.0	888	99.00	
Postoperative complication						
Total	Yes	80	3.56	33	3.68	0.916
	No	2166	96.44	864	96.32	
Bleeding	Yes	29	1.29	17	1.90	0.248
	No	2217	98.71	880	98.10	
Seroma	Yes	51	2.27	14	1.56	0.266
	No	2195	97.73	883	98.44	
Bowell injury/anastomotic leakage	Yes	1	0.04	0	0.00	1.000
	No	2245	99.96	897	100.0	
Wound healing disorders	Yes	2	0.09	4	0.45	0.059
	No	2244	99.91	893	99.55	
Ileus	–	–	–	–	–	–
	No	2246	100.0	897	100.0	
Reoperations	Yes	27	1.20	9	1.00	0.714
	No	2219	98.80	888	99.00	
Recurrence on follow-up	Yes	28	1.25	10	1.11	0.858
	No	2218	98.75	887	98.89	
Pain in rest on follow-up	Yes	133	5.92	78	8.70	0.007
	No	2113	94.08	819	91.30	
Pain on exertion on follow-up	Yes	250	11.13	135	15.05	0.003
	No	1996	88.87	762	84.95	
Pain requiring treatment	Yes	85	3.78	40	4.46	0.419
	No	2161	96.22	857	95.54	

 For multivariable analysis of intraoperative complications, complication-related reoperations and recurrence on 1-year follow-up, it was not possible to calculate any model because of the paucity of relevant cases. The results of the model that explored the variables influencing onset of postoperative complications are illustrated in Table 5 (model matching: *p* = 0.002). Only medial EHS localization impacted the postoperative complication rate. Medial EHS classification reduced the risk of postoperative complications (OR 0.427 [0.213; 0.857]; *p* = 0.017). But there was no evidence of the surgical technique having impacted the postoperative complication rate. The multivariable analysis results of pain at rest are presented in Table 6 (model matching: *p* < 0.001). Here, the BMI proved to be the strongest influence factor (*p* = 0.001). A five-point higher BMI increased the risk of pain at rest (five-point OR 1.351 [1.127; 1.620]). On the other hand, laparo-endoscopic operation (OR 0.643 [0.476; 0.868]; *p* = 0.004) and larger defect size (III vs I: OR 0.500 [0.307; 0.815]; *p* = 0.021) significantly reduced the risk of pain at rest. The multivariable analysis results of pain on exertion are given in Table 7 (model matching: *p* < 0.001). These were highly significantly affected by age and hernia defect size (*p* < 0.001). A higher age (10-year OR 0.825 [0.760; 0.897]) as well as larger hernias (II vs I: OR 0.704 [0.541; 0.916]; III vs I: OR 0.479 [0.331; 0.693]) reduced the risk of pain on exertion. Likewise, laparo-endoscopic operations (OR 0.679 [0.537; 0.857]; *p* = 0.001) compared with open operations reduced the risk for onset of pain on exertion. Similarly, lateral EHS classification reduced the risk (OR 0.624 [0.422; 0.922]; *p* = 0.018) of pain on exertion. However, the risk was increased in association with a five-point higher BMI (five-point OR 1.251 [1.081; 1.449]; *p* = 0.003). The multivariable analysis results of chronic pain requiring treatment are presented in Table 8 (model matching: *p* = 0.005). Here, only the BMI proved to be a significant influence factor (*p* = 0.014). A five-point higher BM increased the rate of pain requiring treatment (five-point OR 1.320 [1.058; 1.647]). However, there was no evidence of the surgical technique having impacted the rate of pain requiring treatment.

**Table 5:** Multivariable analysis of postoperative complications in patients with recurrent inguinal hernia repair according to the guidelines

Parameter	*p* value	Category	OR estimate	95% CI	
EHS-classification medial	0.017	Yes versus no	0.427	0.213	0.857
Age (10-year OR)	0.081		1.148	0.983	1.339
Defect size	0.118	II (1.5–3 cm) versus I (<1.5 cm)	0.848	0.502	1.434
		III (>3 cm) versus I (<1.5 cm)	1.382	0.756	2.526
Risk factors	0.139	Yes versus no	1.371	0.903	2.083
BMI (five-point OR)	0.155		0.807	0.600	1.085
ASA score	0.306	II versus I	0.817	0.486	1.370
		III/IV versus I	1.177	0.600	2.308
EHS-classification lateral	0.372	Yes versus no	0.723	0.354	1.474
EHS-classification femoral	0.647	Yes versus no	1.263	0.466	3.426
Operation	0.772	Endoscopic versus open	0.939	0.616	1.434
EHS-classification scrotal	0.862	Yes versus no	1.121	0.308	4.077

**Table 6:** Multivariable analysis of pain in rest in 1-year follow-up in patients with recurrent inguinal hernia repair according to the guidelines

Parameter	*p* value	Category	OR estimate	95% CI	
BMI (five-point OR)	0.001		1.351	1.127	1.620
Operation	0.004	Endoscopic versus open	0.643	0.476	0.868
Defect size	0.021	II (1.5–3 cm) versus I (<1.5 cm)	0.794	0.562	1.123
		III (>3 cm) versus I (<1.5 cm)	0.500	0.307	0.815
Age (10-year OR)	0.064		0.902	0.809	1.006
EHS-classification lateral	0.087	Yes versus no	0.629	0.370	1.070
EHS-classification medial	0.122	Yes versus no	0.659	0.389	1.118
Risk factor	0.129	Yes versus no	1.278	0.931	1.754
EHS-classification femoral	0.834	Yes versus no	0.913	0.392	2.130
ASA score	0.888	II versus I	0.917	0.643	1.307
		III/IV versus I	0.943	0.552	1.610
EHS-classification scrotal	0.974	Yes versus no	0.000	0.000	I

*I* Infinity

**Table 7:** Multivariable analysis of pain on exertion in 1-year follow-up in patients with recurrent inguinal hernia repair according to the guidelines

Parameter	*p* value	Category	OR estimate	95% CI	
Age (10-year OR)	<0.001		0.825	0.760	0.897
Defect size	<0.001	II (1.5–3 cm) versus I (<1.5 cm)	0.704	0.541	0.916
		III (>3 cm) versus I (<1.5 cm)	0.479	0.331	0.693
Operation	0.001	Endoscopic versus open	0.679	0.537	0.857
BMI (five-point OR)	0.003		1.251	1.081	1.449
EHS-classification lateral	0.018	Yes versus no	0.624	0.422	0.922
EHS-classification scrotal	0.094	Yes versus no	0.178	0.024	1.339
EHS-classification medial	0.180	Yes versus no	0.765	0.517	1.131
Risk factor	0.512	Yes versus no	1.087	0.847	1.393
ASA score	0.764	II versus I	0.981	0.749	1.285
		III/IV versus I	1.114	0.737	1.682
EHS-classification femoral	0.933	Yes versus no	0.973	0.511	1.850

**Table 8:** Multivariable analysis of chronic pain requiring treatment in 1-year follow-up in patients with recurrent inguinal hernia repair according to the guidelines

Parameter	*p* value	Category	OR estimate	95% CI	
BMI (five-point OR)	0.014		1.320	1.058	1.647
EHS-classification lateral	0.051	Yes versus no	0.494	0.243	1.004
Age (10-year OR)	0.053		0.871	0.758	1.002
EHS-classification medial	0.054	Yes versus no	0.501	0.248	1.012
ASA score	0.240	II versus I	1.048	0.654	1.679
		III/IV versus I	1.607	0.834	3.094
Risk factor	0.253	Yes versus no	1.263	0.846	1.886
Operation	0.260	Endoscopic versus open	0.797	0.538	1.182
Defect size	0.294	II (1.5–3 cm) versus I (<1.5 cm)	0.944	0.597	1.493
		III (>3 cm) versus I (<1.5 cm)	0.634	0.338	1.191
EHS-classification femoral	0.476	Yes versus no	1.390	0.561	3.445
EHS-classification scrotal	0.979	Yes versus no	0.000	0.000	I

*I* Infinity

- How do the outcomes of laparo-endoscopic recurrent inguinal hernia repair differ on compliance versus non-compliance with the guidelines?

In the laparo-endoscopic recurrent operation group, the recurrent operation was performed for *n* = 233/2482 (9.4%) patients following laparo-endoscopic primary mesh repair, i.e., not in compliance with the guidelines of the international hernia societies (Table 9). These cases are compared below with the *n* = 2246/2482 (90.6%) patients who were operated on in compliance with the guidelines, with laparo-endoscopic procedure for recurrent repair following previous open primary inguinal hernia operation (Table 9). No significant difference was identified between the two groups with regard to the mean age and BMI (Table 10). The laparo-endoscopic recurrent repairs not conducted in compliance with the guidelines revealed a significantly higher proportion of larger defects as well as a smaller proportion of lateral inguinal hernia recurrences (Table 11). No relevant differences were found for the other variables and risk factors. When recurrent repair was performed as per the guidelines, the laparo-endoscopic procedure was found to be associated with fewer intraoperative (1.2 vs 3.0%; *p* = 0.019) and postoperative complications (3.6 vs 8.6%; *p* < 0.001) as well as a lower re-recurrence risk (1.2 vs 3.4%; *p* = 0.008; Table 12). No differences were identified for the pain rates.

**Table 9:** Laparo-endoscopic unilateral recurrent inguinal hernia repairs on compliance versus non-compliance with the guidelines

	Previous operations						Total	
	Suture		Open mesh		Endoscopic mesh			
	*N*	ColPctN	*N*	ColPctN	*N*	ColPctN	*N*	ColPctN
Guidelines								
No	–	–	–	–	233	100.0	233	9.4
Yes	1528	100.0	718	100.0	–	–	2246	90.6
Total	1528	100.0	718	100.0	233	100.0	2479	100.0

**Table 10:** Age and BMI of patients with laparo-endoscopic unilateral recurrent inguinal hernia repair on compliance versus non-compliance with the guidelines

		Guidelines		*p*
		Yes	No	
Age (years)	Mean ± STD	58.9 ± 15.6	60.1 ± 14.2	0.199
BMI	Mean ± STD	25.9 ± 3.4	26.2 ± 3.0	0.306

**Table 11:** Demographic and surgery-related parameters and risk factors for patients with laparo-endoscopic unilateral recurrent inguinal hernia repair on compliance versus non-compliance with the guidelines

		Guideline				*p*
		Yes		No		
		*n*	%	*n*	%	
ASA score	I	562	24.99	59	25.32	0.992
	II	1303	57.94	134	57.51	
	III/IV	384	17.07	40	17.17	
Defect size	I (<1.5 cm)	419	18.63	34	14.59	0.001
	II (1.5–3 cm)	1460	64.92	139	59.66	
	III (>3 cm)	370	16.45	60	25.75	
Risk factor						
Total	Yes	687	30.55	60	25.75	0.129
	No	1562	69.45	173	74.25	
COPD	Yes	151	6.71	14	6.01	0.681
	No	2098	93.29	219	93.99	
Diabetes	Yes	129	5.74	10	4.29	0.361
	No	2120	94.26	223	95.71	
Aortic aneurism	Yes	16	0.71	1	0.43	0.619
	No	2233	99.29	232	99.57	
Immunosuppression	Yes	14	0.62	1	0.43	0.717
	No	2235	99.38	232	99.57	
Corticoids	Yes	20	0.89	1	0.43	0.465
	No	2229	99.11	232	99.57	
Smoking	Yes	262	11.65	30	12.88	0.580
	No	1987	88.35	203	87.12	
Coagulopathy	Yes	33	1.47	3	1.29	0.827
	No	2216	98.53	230	98.71	
Antiplatelet medication	Yes	202	8.98	15	6.44	0.191
	No	2047	91.02	218	93.56	
Anticoagulation therapy	Yes	44	1.96	4	1.72	0.800
	No	2205	98.04	229	98.28	
EHS-classification medial	Yes	1115	49.58	120	51.50	0.576
	No	1134	50.42	113	48.50	
EHS-classification lateral	Yes	1351	60.07	118	50.64	0.005
	No	898	39.93	115	49.36	
EHS-classification femoral	Yes	77	3.42	6	2.58	0.493
	No	2172	96.58	227	97.42	
EHS-classification scrotal	Yes	27	1.20	5	2.15	0.223
	No	2222	98.80	228	97.85

**Table 12:** Intra- and postoperative compilations, complication-related reoperations and 1-year follow-up-results of patients with laparo-endoscopic unilateral recurrent inguinal hernia repair on compliance versus non-compliance with the guidelines

		Guidelines				*p*
		Yes		No		
		*n*	%	*n*	%	
Intraoperative complication						
Total	Yes	26	1.16	7	3.00	0.019
	No	2223	98.84	226	97.00	
Bleeding	Yes	15	0.67	7	3.00	<0.001
	No	2234	99.33	226	97.00	
Injury						
Total	Yes	17	0.76	3	1.29	0.388
	No	2232	99.24	230	98.71	
Vascular	Yes	8	0.36	3	1.29	0.042
	No	2241	99.64	230	98.71	
Bowell	Yes	5	0.22	0	0.00	0.471
	No	2244	99.78	233	100.0	
Bladder	Yes	2	0.09	0	0.00	0.649
	No	2247	99.91	233	100.0	
Postoperative complication						
Total	Yes	80	3.56	20	8.58	<0.001
	No	2169	96.44	213	91.42	
Bleeding	Yes	29	1.29	6	2.58	0.113
	No	2220	98.71	227	97.42	
Seroma	Yes	51	2.27	14	6.01	<0.001
	No	2198	97.73	219	93.99	
Infection	Yes	1	0.04	0	0.00	0.748
	No	2248	99.96	233	100.0	
Bowell injury	Yes	1	0.04	0	0.00	0.748
	No	2248	99.96	233	100.0	
Wound healing disorders	Yes	1	0.04	0	0.00	0.748
	No	2248	99.96	233	100.0	
Reoperations	Yes	27	1.20	6	2.58	0.081
	No	2222	98.80	227	97.42	
Recurrence on follow-up	Yes	28	1.24	8	3.43	0.008
	No	2221	98.76	225	96.57	
Pain in rest on follow-up	Yes	133	5.91	20	8.58	0.107
	No	2116	94.09	213	91.42	
Pain on exertion on follow-up	Yes	250	11.12	34	14.59	0.113
	No	1999	88.88	199	85.41	
Pain requiring treatment on follow-up	Yes	85	3.78	10	4.29	0.698
	No	2164	96.22	223	95.71	

For multivariable analysis of the intraoperative complications, complication-related reoperations and re-recurrences, it was not possible to calculate a valid model on differences of follow-up because of the small number of positive cases. On univariable analysis of pain at rest, pain on exertion and chronic pain requiring treatment, no difference was discerned for the procedures conducted in accordance with the guidelines.

The multivariable analysis results for the postoperative complications are presented in Table 13 (model matching: *p* < 0.001). The postoperative complications were impacted, in particular, by the procedures conducted in accordance with the guidelines (*p* = 0.001). When the guidelines were observed, the risk of onset of postoperative complications declined (OR 0.419 [0.248; 0.708]; *p* = 0.001). Besides, the defect size had a significant effect on the postoperative complication risk. Larger hernia defects (III vs I: OR 2.329 [1.135; 4.779]; *p* = 0.018) were associated with a higher complication risk.

**Table 13:** Multivariable analysis of postoperative complications in patients with laparo-endoscopic unilateral recurrent inguinal hernia repair

Parameter	*p* value	Category	OR estimate	95% CI	
Guidelines	0.001	Yes versus no	0.419	0.248	0.708
Defect size	0.018	II (1.5–3 cm) versus I (<1.5 cm)	1.256	0.656	2.404
		III (>3 cm) versus I (<1.5 cm)	2.329	1.135	4.779
Age (10-year OR)	0.089		1.152	0.979	1.357
EHS-classification medial	0.115	Yes versus no	0.572	0.285	1.146
Risk factor	0.269	Yes versus no	1.293	0.820	2.038
BMI (five-point OR)	0.420		0.876	0.634	1.210
EHS-classification femoral	0.429	Yes versus no	1.485	0.558	3.953
EHS-classification lateral	0.532	Yes versus no	0.797	0.392	1.621
EHS-classification scrotal	0.612	Yes versus no	1.378	0.399	4.758
ASA score	0.657	II versus I	0.849	0.484	1.489
		III/IV versus I	1.056	0.512	2.179

3b. How do the outcomes of open recurrent inguinal hernia repair differ on compliance versus non-compliance with the guidelines?

In the open recurrent repair group, only *n* = 897/2.320 (38.5%) of operations were performed following previous primary laparo-endoscopic inguinal hernia repair, i.e., according to the guidelines. Conduct of open recurrent repair following previous suture procedure for the primary inguinal hernia repair (*n* = 1.011/2.320; 43.4%) and after mesh procedure (*n* = 412/2.320; 17.7%) was not in compliance with the guidelines (Table 14). Below are now compared the open recurrent inguinal hernia repair procedures conducted on compliance (*n* = 897/2.320; 38.5%) versus non-compliance with the guidelines (*n* = 1.423/2.320; 61.3%).

**Table 14:** Open unilateral recurrent inguinal hernia repairs on compliance versus non-compliance with the guidelines

	Previous operations						Total	
	Suture		Open mesh		Endoscopic mesh			
	*N*	ColPctN	*N*	ColPctN	*N*	ColPctN	*N*	ColPctN
Guidelines								
No	1011	100.0	412	100.0	–	–	1423	61.3
Yes	–	–	–	–	897	100.0	897	38.7
Total	1011	100.0	412	100.0	897	100.0	2320	100.0

Patients with recurrent inguinal hernias repaired in accordance with the guidelines had a significantly lower age and higher BMI (Table 15). Furthermore, patients operated on with an open procedure as per the guidelines had a significantly lower ASA score, smaller hernia defects, fewer risk factors and fewer lateral and scrotal hernias (Table 16). When the recurrent repair was performed as per the guidelines, open repair was associated with fewer postoperative complications (3.6 vs 5.8%; *p* = 0.021) and complication-related reoperation (1.0 vs 2.1%; *p* = 0.041) as well as a lower re-recurrence risk (1.1 vs 2.6%; *p* = 0.012). On the other hand, there was an increase in the risk of pain at rest (8.6 vs 5.4%; *p* = 0.003) and on exertion (15.0 vs 10.2%; *p* < 0.001; Table 17).

**Table 15:** Age and BMI of patients with open unilateral recurrent inguinal hernia repair on compliance versus non-compliance with the guidelines

		Guidelines		*p*
		Yes	No	
Age (years)	Mean ± STD	59.3 ± 13.5	62.5 ± 16.2	<0.001
BMI	Mean ± STD	26.3 ± 3.6	25.8 ± 3.4	<0.001

**Table 16:** Demographic and surgery-related parameters and risk factors for patients with open unilateral recurrent inguinal hernia repair on compliance versus non-compliance with the guidelines

		Guidelines				*p*
		Yes		No		
		*n*	%	*n*	%	
ASA score	I	258	28.45	368	25.86	<0.001
	II	509	56.12	708	49.75	
	III/IV	140	15.44	347	24.39	
Defect size	I (<1.5 cm)	154	16.98	240	16.87	0.028
	II (1.5–3 cm)	498	54.91	711	49.96	
	III (>3 cm)	255	28.11	472	33.17	
Risk factor						
Total	Yes	277	30.54	559	39.28	<0.001
	No	630	69.46	864	60.72	
COPD	Yes	67	7.39	149	10.47	0.012
	No	840	92.61	1274	89.53	
Diabetes	Yes	51	5.62	114	8.01	0.028
	No	856	94.38	1309	91.99	
Aortic aneurism	Yes	4	0.44	11	0.77	0.329
	No	903	99.56	1412	99.23	
Immunosuppression	Yes	10	1.10	23	1.62	0.306
	No	897	98.90	1400	98.38	
Corticoid	Yes	8	0.88	29	2.04	0.030
	No	899	99.12	1394	97.96	
Smoking	Yes	111	12.24	203	14.27	0.162
	No	796	87.76	1220	85.73	
Coagulopathy	Yes	9	0.99	40	2.81	0.003
	No	898	99.01	1383	97.19	
Antiplatelet medication	Yes	79	8.71	186	13.07	0.001
	No	828	91.29	1237	86.93	
Anticoagulation therapy	Yes	25	2.76	50	3.51	0.313
	No	882	97.24	1373	96.49	
EHS-classification medial	Yes	523	57.66	795	55.87	0.394
	No	384	42.34	628	44.13	
EHS-classification lateral	Yes	460	50.72	800	56.22	0.009
	No	447	49.28	623	43.78	
EHS-classification femoral	Yes	15	1.65	32	2.25	0.319
	No	892	98.35	1391	97.75	
EHS-classification scrotal	Yes	12	1.32	63	4.43	<0.001
	No	895	98.68	1360	95.57

**Table 17:** Intra- and postoperative complications, complication-related reoperations and 1-year follow-up results of patients with open unilateral recurrent inguinal hernia repair on compliance versus non-compliance with the guidelines

		Yes		No		*p*
		*n*	%	*n*	%	
Intraoperative complication						
Total	Yes	14	1.54	23	1.62	0.891
	No	893	98.46	1400	98.38	
Bleeding	Yes	3	0.33	12	0.84	0.131
	No	904	99.67	1411	99.16	
Injury						
Total	Yes	12	1.32	14	0.98	0.447
	No	895	98.68	1409	99.02	
Vascular	Yes	0	0.00	3	0.21	0.166
	No	907	100.0	1420	99.79	
Bowell	Yes	0	0.00	4	0.28	0.110
	No	907	100.0	1419	99.72	
Bladder	Yes	1	0.11	1	0.07	0.748
	No	906	99.89	1422	99.93	
Nerve	Yes	9	0.99	1	0.07	<0.001
	No	898	99.01	1422	99.93	
Postoperative complication						
Total	Yes	33	3.64	82	5.76	0.021
	No	874	96.36	1341	94.24	
Bleeding	Yes	17	1.87	45	3.16	0.060
	No	890	98.13	1378	96.84	
Seroma	Yes	14	1.54	30	2.11	0.329
	No	893	98.46	1393	97.89	
Infection	Yes	0	0.00	3	0.21	0.166
	No	907	100.0	1420	99.79	
Wound healing disorders	Yes	4	0.44	7	0.49	0.861
	No	903	99.56	1416	99.51	
Reoperation	Yes	9	0.99	30	2.11	0.041
	No	898	99.01	1393	97.89	
Recurrence on follow-up	Yes	10	1.10	37	2.60	0.012
	No	897	98.90	1386	97.40	
Pain in rest on follow-up	Yes	78	8.60	77	5.41	0.003
	No	829	91.40	1346	94.59	
Pain on exertion on follow-up	Yes	136	14.99	145	10.19	<0.001
	No	771	85.01	1278	89.81	
Pain requiring treatment on follow-up	Yes	40	4.41	50	3.51	0.274
	No	867	95.59	1373	96.49	

For multivariable analysis of the intraoperative complications, complication-related reoperations and re-recurrences, it was not possible to calculate a valid model since the number of positive cases was too small. Univariable analysis of chronic pain requiring treatment did not detect any difference for repair as per the guidelines; therefore, no multivariable model was calculated.

The multivariable analysis results of variables influencing onset of postoperative complications are given in Table 18 (model matching: *p* = 0.002).

**Table 18:** Multivariable analysis of postoperative complications in patients with open unilateral recurrent inguinal hernia repair

Parameter	*p* value	Category	OR estimate	95% CI	
Age (10-year OR)	0.003		1.275	1.085	1.498
Risk factor	0.118	Yes versus no	1.390	0.919	2.102
Guidelines	0.155	Yes versus no	0.734	0.479	1.124
EHS-classification lateral	0.165	Yes versus no	0.654	0.359	1.191
Defect size	0.181	II (1.5–3 cm) versus I (<1.5 cm)	0.718	0.420	1.225
		III (>3 cm) versus I (<1.5 cm)	1.053	0.600	1.848
EHS-classification medial	0.225	Yes versus no	0.685	0.372	1.262
BMI (five-point OR)	0.392		0.880	0.656	1.180
ASA score	0.434	II versus I	0.742	0.439	1.256
		III/IV versus I	0.913	0.470	1.775
EHS-classification femoral	0.935	Yes versus no	0.950	0.276	3.275
EHS-classification scrotal	0.975	Yes versus no	0.985	0.371	2.612

The postoperative complications were only affected by age, with older patients (10-year OR 1.275 [1.085; 1.498]; *p* = 0.003) having a higher risk of postoperative complications. There was no evidence that repair as per the guidelines impacted the postoperative complications.

The multivariable analysis results for pain at rest are presented in Table 19 (model matching: *p* < 0.001). Here, the hernia defect size proved to be the strongest influence factor (*p* = 0.006). A larger recurrent hernia (II vs I: OR 0.521 [0.346; 0.786]; III vs I: OR 0.560 [0.352; 0.892]) reduced the risk of pain at rest.

**Table 19:** Multivariable analysis of pain at rest in patients with open unilateral recurrent inguinal hernia repair

Parameter	*p* value	Category	OR estimate	95% CI	
Defect size	0.006	II (1.5–3 cm) versus I (<1.5 cm)	0.521	0.346	0.786
		III (>3 cm) versus I (<1.5 cm)	0.560	0.352	0.892
Guidelines	0.016	Yes versus no	1.508	1.079	2.107
BMI (five-point OR)	0.019		1.295	1.043	1.609
Age (10-year OR)	0.110		0.902	0.795	1.023
EHS-classification femoral	0.164	Yes versus no	0.238	0.032	1.798
EHS-classification lateral	0.243	Yes versus no	0.716	0.409	1.254
EHS-classification medial	0.352	Yes versus no	0.761	0.428	1.353
ASA score	0.490	II versus I	0.829	0.556	1.236
		III/IV versus I	0.697	0.375	1.295
Risk factor	0.528	Yes versus no	1.126	0.779	1.628
EHS-classification scrotal	0.756	Yes versus no	0.839	0.276	2.545

Likewise, repair as per the guidelines (*p* = 0.016) and BMI (*p* = 0.019) had a significant influence on pain at rest. Repair as per the guidelines (OR 1.508 [1.079; 2.107]) as well as a five-point higher BMI (five-point OR 1.295 [1.043; 1.609]) increased the risk of pain at rest.

Another descriptive analysis revealed that the increased risk of pain at rest was attributed primarily to the small-sized (<1.5 cm) and medium-sized (1.5–3 cm) hernias (Table 20).

**Table 20:** Correlation of the defect size, compliance versus non-compliance with the guidelines and pain in rest on follow-up in patients with open unilateral recurrent inguinal hernia repair

		Defect size						All	
		I (<1.5 cm)		II (1.5–3 cm)		III (>3 cm)			
		*N*	%	*N*	%	*N*	%	*N*	%
Guidelines	Pain in rest on follow-up								
No	No	217	90.4	685	96.3	444	94.1	1346	94.6
	Yes	23	9.6	26	3.7	28	5.9	77	5.4
Yes	No	135	87.7	455	91.4	239	93.7	829	91.4
	Yes	19	12.3	43	8.6	16	6.3	78	8.6

The multivariable analysis results for pain on exertion are illustrated in Table 21 (model matching: *p* < 0.001). These were significantly influenced by the hernia defect size (*p* = 0.002), repair as per the guidelines (*p* = 0.010), BMI (*p* = 0.023), age (*p* = 0.027) and scrotal EHS classification (*p* = 0.036). A higher age (10-year OR 0.897 [0.814; 0.988]), larger hernias (II vs I: OR 0.654 [0.475; 0.901]; III vs I: OR 0.517 [0.335; 0.754]) as well as scrotal EHS classification (OR 0.211 [0.049; 0.900]) reduced the risk of pain on exertion. Conversely, there was a higher risk of pain for repair as per the guidelines (OR 1.401 [1.084; 1.810]) and for a five-point larger BMI (five-point OR 1.224 [1.029; 1.456]). Likewise, for pain on exertion the risk was attributable, in particular, to small-sized (<1.5 cm) and medium-sized (1.5–3 cm) recurrent hernias (Table 22).

**Table 21:** Multivariable analysis of pain on exertion in patients with open unilateral recurrent inguinal hernia repair

Parameter	*p* value	Category	OR estimate	95% CI	
Defect size	0.002	II (1.5–3 cm) versus I (<1.5 cm)	0.654	0.475	0.901
		III (>3 cm) versus I (<1.5 cm)	0.517	0.355	0.754
Guidelines	0.010	Yes versus no	1.401	1.084	1.810
BMI (five-point OR)	0.023		1.224	1.029	1.456
Age (10-year OR)	0.027		0.897	0.814	0.988
EHS-classification scrotal	0.036	Yes versus no	0.211	0.049	0.900
EHS-classification lateral	0.054	Yes versus no	0.653	0.423	1.007
Risk factor	0.241	Yes versus no	1.182	0.894	1.563
EHS-classification femoral	0.247	Yes versus no	0.531	0.182	1.551
EHS-classification medial	0.292	Yes versus no	0.787	0.504	1.229
ASA score	0.715	II versus I	1.054	0.769	1.446
		III/IV versus I	0.905	0.563	1.453

**Table 22:** Correlation of the defect size, compliance versus non-compliance with the guidelines and pain on exertion on follow-up in patients with open unilateral recurrent inguinal hernia repair

		Defect size						All	
		I (<1.5 cm)		II (1.5–3 cm)		III (>3 cm)			
		*N*	%	*N*	%	*N*	%	*N*	%
Guidelines	Pain on exertion on follow-up								
No	No	204	85.0	644	90.6	430	91.1	1278	89.8
	Yes	36	15.0	67	9.4	42	8.9	145	10.2
Yes	No	121	78.6	421	84.5	229	89.8	771	85.0
	Yes	33	21.4	77	15.5	26	10.2	136	15.0

## Discussion

1. The present analysis of data from the Herniamed Registry [29] first investigated to what extent participants in the Herniamed Hernia Registry [29] complied with the recommendations set out in the guidelines of the European Hernia Society (EHS). This revealed that laparo-endoscopic recurrent repair was used in 61.6% of cases following previous open suture repair and in 28.9% cases following open mesh repair as well as in 9.4% of cases following previous laparo-endoscopic operations. Hence, more than 90% of laparo-endoscopic recurrent repair procedures were performed in accordance with the EHS guidelines. Only 9.4% did not comply with the guidelines.

Matters were different for open recurrent repair. Only 38.5% of open recurrent repair operations were conducted following primary laparo-endoscopic repair. 43.4% of open recurrent repair procedures were performed following previous open suture repair and 17.7% following previous open mesh repair. As such, more than 60% of open recurrent operations did not comply with the recommendations of the guidelines. Already Richards et al. [13] and Richards and Earnshaw [28] pointed out that surgeons using predominantly open hernia surgery techniques also use predominantly open surgery for recurrent repair. It appears that the guidelines, which were first published in 2009 [23], have not changed that scenario. Further high-quality studies are needed to demonstrate that repair as per the guidelines really does achieve a better outcome for patients. Only when convincing evidence based on high-quality trials is available can greater acceptance of the guidelines be expected. Since to date no such studies have been carried out, it is no surprise that surgeons have called upon their own expertise when deciding on the surgical technique used to treat patients with recurrent inguinal hernia. Guidelines always only reflect the current state of knowledge gained from the studies reported in the scientific literature. If new published data are added, the recommendations may also change. Mere deviation from a guideline is unlikely to be considered as malpractice in litigation, unless the practice concerned is so well established that no responsible surgeon would fail to adhere to it [31].

2. To date, no study has compared the outcomes of recurrent inguinal hernia repair carried out in compliance with the guidelines. Therefore, the present analysis of Herniamed data [29] compared laparo-endoscopic with open recurrent repair performed as per the guidelines. No significant difference was identified between laparo-endoscopic and open techniques performed as per the guidelines in terms of the overall intraoperative complication rate, postoperative complication rate, complication-related reoperation rate, recurrence rate and rate of chronic pain requiring treatment. However, with regard to the intraoperative complications open recurrent repair was associated with significantly more nerve injuries as well as more pain at rest and pain on exertion on 1-year follow-up.

Multivariable analysis confirmed that laparo-endoscopic repair had a significant impact on pain at rest and pain on exertion, and was associated with a lower pain rate compared with open recurrent repair. Even on compliance with the guidelines, a significantly higher rate of pain at rest and pain on exertion must be expected when open repair is used following previous laparo-endoscopic operations compared with laparo-endoscopic repair after previous open repair. Therefore, such recurrent repair operations should be performed by surgeons who are highly experienced in the respective technique. Therefore, despite observance of the guidelines, higher rates of pain at rest and pain on exertion must be expected on using open recurrent repair following primary laparo-endoscopic repair than when using laparo-endoscopic recurrent repair following primary open repair.

3. In particular, since a large number of open (61.1%) and also a smaller number of laparo-endoscopic (9.4%) recurrent repair procedures were not performed in accordance with the recommendations of the guidelines, the question arises as to how the outcomes compare with the respective repair procedures carried out in compliance with the guidelines.

If recurrent repair is conducted as per the guidelines, laparo-endoscopic repair is associated with fewer intraoperative and postoperative complications and with a lower re-recurrence rate. No difference was found for the pain rates. Multivariable analysis demonstrated especially for the postoperative complications the impact of repair as per the guidelines.

Comparison of open recurrent repair conducted on compliance versus non-compliance with the guidelines revealed fewer postoperative complications and complication-related reoperation rates as well as a lower re-recurrence rate following repair as per the guidelines. On the other hand, the risk of pain at rest and on exertion was higher on compliance with the guidelines. Multivariable analysis revealed that the postoperative complications were only affected by age but not by the use of a repair procedure in accordance with the guidelines. Matters were different for pain at rest and pain on exertion. For the latter, multivariable analysis confirmed that repair as per the guidelines exerted a significantly negative effect on onset of pain at rest and pain on exertion. However, multivariable analysis as well as an additional analysis demonstrated that a small defect size had the greatest impact on the risk of pain at rest and pain on exertion. Likewise, a higher BMI negatively impacted the risk of pain at rest and pain on exertion. Although recommended in the guidelines, patients with a small defect size and a higher BMI have a higher risk of pain at rest and exertion following open repair of a recurrence after a previous laparo-endoscopic inguinal hernia repair. Therefore, sufficient diagnostic work-up of a small recurrence as cause of groin pain is mandatory.

In summary, it can be stated that in the Herniamed Registry (1) 90% of the laparo-endoscopic and only 40% of open recurrent inguinal hernia repair operations are carried out in accordance with the EHS guidelines; (2) comparison of laparo-endoscopic with open recurrent repair conducted in accordance with the guidelines demonstrated that open recurrent repair as per the guidelines was associated with a higher risk of pain at rest and pain on exertion on 1-year follow-up; and (3) finally, comparison of recurrent repair procedures on compliance versus non-compliance with the guidelines showed that both laparo-endoscopic and open repair operations that did not comply with the guidelines presented a higher risk of perioperative complications and re-recurrences. As such, the recommendations set out in the EHS guidelines should be implemented, but considering the specific circumstances of a given patient.
